# Immunomonitoring of Stage IV Relapsed Neuroblastoma Patients Undergoing Haploidentical Hematopoietic Stem Cell Transplantation and Subsequent GD2 (ch14.18/CHO) Antibody Treatment

**DOI:** 10.3389/fimmu.2021.690467

**Published:** 2021-07-22

**Authors:** Christian Martin Seitz, Tim Flaadt, Markus Mezger, Anne-Marie Lang, Sebastian Michaelis, Marie Katz, Desireé Syring, Alexander Joechner, Armin Rabsteyn, Nikolai Siebert, Sascha Troschke-Meurer, Maxi Zumpe, Holger N. Lode, Sile F. Yang, Daniel Atar, Anna-Sophia Mast, Sophia Scheuermann, Florian Heubach, Rupert Handgretinger, Peter Lang, Patrick Schlegel

**Affiliations:** ^1^Department of Pediatric Hematology and Oncology, University Children’s Hospital, Eberhard Karls University Tuebingen, Tuebingen, Germany; ^2^Cluster of Excellence iFIT (EXC 2180) “Image-Guided and Functionally Instructed Tumor Therapies”, University of Tuebingen, Tuebingen, Germany; ^3^Cellular Cancer Therapeutics Unit, Children’s Medical Research Institute, Westmead, NSW, Australia; ^4^Department of Pediatric Hematology and Oncology, University Medicine Greifswald, Greifswald, Germany; ^5^School of Medical Sciences, Faculty of Medicine and Health, University of Sydney, Sydney, NSW, Australia; ^6^Department of Pediatric Hematology and Oncology, Westmead Children’s Hospital, Westmead, NSW, Australia

**Keywords:** neuroblastoma, immunomonitoring immunotherapy, GD2 antibody therapy, haploidentical allogeneic stem cell transplantation, antibody-dependent cellular cytotoxicity, complement-dependent cytotoxicity

## Abstract

Haploidentical stem cell transplantation (haplo SCT) in Stage IV neuroblastoma relapsed patients has been proven efficacious, while immunotherapy utilizing the anti-GD2 antibody dinutuximab beta has become a standard treatment for neuroblastoma. The combinatorial therapy of haplo SCT and dinutuximab may potentiate the efficacy of the immunotherapy. To gain further understanding of the synergistic effects, functional immunomonitoring was assessed during the clinical trial CH14.18 1021 Antibody and IL2 After haplo SCT in Children with Relapsed Neuroblastoma (NCT02258815). Rapid immune reconstitution of the lymphoid compartment was confirmed, with clinically relevant dinutuximab serum levels found in all patients over the course of treatment. Only one patient developed human anti-chimeric antibodies (HACAs). In-patient monitoring revealed highly functional NK cell posttransplant capable of antibody-dependent cellular cytotoxicity (ADCC). Degranulation of NK cell subsets revealed a significant response increased by dinutuximab. This was irrespective of the KIR receptor–ligand constellation within the NK subsets, defined by the major KIR receptors CD158a, CD158b, and CD158e. Moreover, complement-dependent cytotoxicity (CDC) was shown to be an extremely potent effector-cell independent mechanism of tumor cell lysis, with a clear positive correlation to GD2 expression on the cancer cells as well as to the dinutuximab concentrations. The *ex vivo* testing of patient-derived effector cells and the sera collected during dinutuximab therapy demonstrated both high functionality of the newly established lymphoid immune compartment and provided confidence that the antibody dosing regimen was sufficient over the duration of the dinutuximab therapy (up to nine cycles in a 9-month period). During the course of the dinutuximab therapy, proinflammatory cytokines and markers (sIL2R, TNFa, IL6, and C reactive protein) were significantly elevated indicating a strong anti-GD2 immune response. No impact of FcGR polymorphism on event-free and overall survival was found. Collectively, this study has shown that in-patient functional immunomonitoring is feasible and valuable in contributing to the understanding of anti-cancer combinatorial treatments such as haplo SCT and antibody immunotherapy.

## Introduction

Neuroblastoma is the most common pediatric extracranial solid cancer, accounting for 12% of childhood cancer deaths (1). It arises from cells of the sympathetic nervous system (2). In high-risk neuroblastoma, defined by the presence of metastatic diseases in children older than 12 or 18 months or the MYCN amplification (MNA) in patients of any age, the prognosis is especially dismal, with a 5-year survival of only 40% (3). The current standard therapy consists of a multimodal treatment approach that encompasses a surgical resection or a biopsy, an intensive course of high-dose chemotherapy (six cycles), and another surgical intervention with complete resection of the primary tumor if possible. In addition, tumors with diagnostic ^123^I-meta-iodobenzylguanidine (^123^I-mIBG) uptake may receive a ^131^I-meta-iodobenzylguanidine (^131^I-mIBG) targeted radiation therapy prior to subsequent autologous stem-cell rescue as consolidation therapy and isotretinoin for minimal residual disease (MRD) therapy (4–6). In recent years, immunotherapy has demonstrated promising clinical efficacy. Monoclonal antibodies (mAbs) against the disialoganglioside GD2, an antigen highly expressed on most neuroblastoma cells, with a much lower expression on physiological human tissues including neurons, skin melanocytes, and peripheral sensory nerve fibers, have been developed and intensively studied in clinical trials (7, 8). The chimeric antibody ch14.18, dinutuximab, in combination with granulocyte–macrophage colony-stimulating factor (GM-CSF) or interleukin-2 (IL2) has significantly improved the 2-year event-free survival by 20% and the overall survival by 10% compared to standard therapy in high-risk patients (9). Substantial toxicities observed included pain, fever, allergic reactions, and capillary leak syndrome, which were in part attributed to the use of GM-CSF and IL2. In Europe, ch14.18 was re-cloned in Chinese Hamster Ovary (CHO) cells and designated as ch14.18/CHO (dinutuximab beta) to reflect the molecular difference in the glycosylation pattern compared to ch14.18. In a multicenter, randomized, phase 3 trial (HR-NBL1/SIOPEN), dinutuximab beta has shown to improve the 5-year event-free survival rate by 15% and overall survival by 14% over a historic cohort (10, 11). The results of this trial contributed to the approval of dinutuximab beta in the European Union for the treatment of neuroblastoma. In contrast to MRD settings, the clinical activity of dinutuximab beta has only been demonstrated in combination with high dose IL2 against relapsed and refractory diseases (12), while the single agent activity of dinutuximab beta has not been addressed in clinical trials yet. We have demonstrated that antibody-dependent cell-mediated cytotoxicity (ADCC) by NK cells significantly contributes to the clinical activity and improved event-free survival in patients treated with dinutuximab beta (13). However, repetitive high-dose cytotoxic therapy and tumor-mediated immune editing may lead to dysfunctional immune cells, devoid of therapeutic activity of dinutuximab beta in heavily pretreated neuroblastoma patients. We have shown that haploidentical stem cell transplantation (haplo SCT) utilizing CD3/CD19 depleted G-CSF-mobilized peripheral blood stem cell grafts is a feasible strategy to establish a novel and functional cellular immune system (14). Notably, NK cells were demonstrated as the predominant immune cell population in the early phase of immune reconstitution after haplo SCT. Together, these findings led to the rationale to initiate the “phase I/II feasibility study using ch14.18/CHO antibody and subcutaneous interleukin 2 after haploidentical stem cell transplantation in children with relapsed neuroblastoma” (NCT02258815). Here, we report on the results of the immune monitoring and evaluate the cooperative activity of immune reconstitution and targeted redirection by dinutuximab beta.

## Patients, Materials, and Methods

### Ethical Statement

All procedures involving human participants were in accordance with the ethical standards of the institutional and national research committees, competent authorities, and the 1964 Helsinki declaration and its later amendments or comparable ethical standards. The treatment was conducted according to the clinical trial “CH14.18 1021 Antibody and IL2 After Haplo SCT in Children With Relapsed Neuroblastoma” registered at ClinicalTrials.gov (NCT02258815). Informed consent was obtained from all individual participants or their parents or legal guardians.

### Cell Lines and Culturing Conditions

All cell lines including LAN-1 (ACC 655), LS (ACC 675), SK-N-AS (CRL2137), SH-SY5Y (CRL-2266) were purchased from ATCC or DSMZ (LS) and maintained in complete RPMI 1640 (Biochrom) media or DMEM (Gibco) supplemented with 10–20% of heat-inactivated fetal bovine serum (Biochrom), 2 mM L-glutamine, and 1 mM sodium pyruvate (Biochrom) according to cell culturing instructions. All media contained 100 units/ml of penicillin and 100 µg/ml of streptomycin (Biochrom).

### Flow Cytometry

#### Monitoring of Lymphoid Immune Compartment and Activation State of NK Cells

For the calculation of absolute cell numbers per µl, differential blood counts of patients were measured on ADVIA^®^120-Siemens in the routine hematologic laboratory and calculated accordingly. Reconstitution of lymphocytes and the activation state of NK cells were monitored by flow cytometry using three different combinations of antibodies. BD Multitest™ T cells: CD3^+^ FITC (clone SK7), CD45^+^ PerCp (clone 2D1), CD4^+^ APC (clone SK3), and CD8^+^ PE (clone SK1). BD Multitest™ NK/B cells: CD3^+^ FITC (clone SK7), CD45^+^ PerCp (clone 2D1), CD56/CD16^+^ PE (clones NCAM16.2/B73.1), CD19^+^ APC (clone SJ25C1). Non-activated NK cells were defined as CD56^+^CD16^+^CD69***^−^***; activated NK cells were identified by CD56^+^CD16^+^CD69^+^ immunophenotype using the combination of CD3^+^ FITC (clone SK7), CD45^+^ PerCp (clone 2D1), CD56/CD16^+^ PE (clones NCAM16.2/B73.1), and CD69^+^ APC (clone FN50). Data acquisition was performed by the stem cell laboratory of the GMP facility (University of Tuebingen) using standard protocols. A total of >10,000 cell events were acquired on a BD FACSCalibur™ and analyzed by the CELLQuest™ software.

#### CD107a-Based Degranulation Assay

The frequency of degranulation by NK cells within the PBMCs was quantitated by multi-parameter flow cytometry after 6 h incubation at 37°C and 5% CO_2_ in a Heracell incubator. RPMI 1640 (Biochrom) cell culture media containing 10% FBS (Gibco), 2 mM L-glutamine (Biochrom) were used. Effector cells (PBMCs) cultivated without target cells were defined as a negative control for background activation. Cells stimulated with phorbol-12-myristate-13-acetate (PMA) at 200 ng/ml and ionomycin at 4 µM (Sigma) served as an internal positive control. In the testing conditions, PBMCs were stimulated by coincubation with indicated tumor cell lines with and without GD2-mAb ch14.18/CHO at 1 µg/ml.

CD107a-APC (clone H4A3) antibody (Biolegend) was added directly to the tubes. Monensin (Golgi-Stop, BD Biosciences) was added at a final concentration of 10 µg/ml. According to a standard protocol, PBMCs were stained with CD3 PerCP (SK7), CD56-PECy7 (clone HCD65), CD16 AF700 (clone 3G8), CD158b PE (clone DX27), and CD158e BV421 (clone DX9) (all these antibodies are from Biolegend) as well as CD158a FITC (clone HP-3E4, BD Biosciences) for 10 min at 4°C. After washing, cells were resuspended in 0.5% paraformaldehyde (Sigma) until multi-color flow cytometric analysis was performed on a LSRII instrument (BD Biosciences). A total of >50,000 cell events were acquired and analyzed using FlowJo 10.7.1 software.

#### GD2 Antigen Expression Screening on Neuroblastoma Cell Lines

GD2 expression was measured on a BD™ LSR II flow cytometer using primary labeled GD2 PE (clone 14G2a) mAb and a mouse IgG2a, k PE mAb as isotype control. Antibody staining was done according to standard operating procedure at 4°C in PBS buffer. Staining of tumor cells using primary labeled mAbs compared to isotype control defined antigen positivity. The Median Fluorescence Intensity Ratio (MFIR) was calculated by MFI GD2 PE mAb divided by MFI IgG2a, k PE mAb. Data analyses were performed using FlowJo 10.7.1 software.

### Analysis of Antibody Serum-Levels

Validated detection of ch14.18/CHO in patient samples was performed using the triple-ELISA strategy (limit of detection in serum samples: 58 ng/ml ch14.18/CHO) as previously described (15, 16). The anti-idiotype mAb ganglidiomab (17) was used as capture mAb. Briefly, patient serum samples were first analyzed using the “low sensitivity” ELISA with a detection range of 3.0–25 µg/ml ch14.18/CHO. Then, samples containing ch14.18/CHO levels lower than 3 µg/ml were subjected to reanalysis with the “intermediate sensitivity” ELISA (detection range: 0.5–3.1 µg/ml). Finally, samples with ch14.18/CHO concentrations below 0.5 µg/ml were reanalyzed with the “high sensitivity” ELISA with a detection range of 0.058–1.0 µg/ml (18).

### Analysis of Human Anti-Chimeric Antibody

To analyze HACA development in patients treated with ch14.18/CHO, a validated ELISA allowing specific detection of anti-ch14.18/CHO Ab in patient serum was performed as previously described (19).

### Quantification of Cytokine Levels IL2, IL6, TNFα, And C-Reactive Protein in Patient Serum

For determination of secreted cytokines, patient samples were collected at indicated time points. The quantification was performed by the institute for clinical chemistry and laboratory medicine according to high-standard pharmaceutical protocols undergoing external validation.

### Analysis of NK-cell Activity Cytotoxicity

The cytolytic activity of patient PBMCs was analyzed in a 2h-DELFIA-EuTDA cytotoxicity assay (PerkinElmer/USA) according to the manufacturer’s instructions. A PBMC to neuroblastoma cell line ratio (E:T ratio) 5:1 with or without GD2-mAb-ch14.18 at 1 μg/ml was used. Experiments were analyzed in triplicates using six replicate wells for maximum release (target cells treated for 20 s with ultrasonic homogenizer). The specific lysis was calculated according to the formula: (test release − negative control release)/(maximum release − negative control release) × 100%. The fluorescence intensities were measured on a VICTOR-II-multi-label-reader (Wallac/Finland) as described previously (20).

#### Generation Lentiviral Vectors Encoding Firefly Luciferase-mCherry/GFP

Lentivirus (LV) was produced in Lenti-X™ 293T (Clontech) after lipofection (Lipofectamine 3000, Thermo Fisher) of a second generation packaging plasmid, a VSV-G envelope plasmid and the indicated transfer plasmid. LV containing supernatants were concentrated using the Lenti-X concentrator (TaKaRa) and cryopreserved.

#### Generation of Luciferase Expressing Cell Lines

Transfer plasmids, based on a third generation lentiviral vector plasmid, containing firefly luciferase and mCherry or GFP were kindly provided by Irmela Jeremias, Helmholtz Center Munich, Germany (21). LV particles were generated as described above. Cell lines were transduced at a MOI of three. Transgene expression was confirmed by flow cytometry using the co-expressed fluorescent protein. Transduced cells were enriched by bulk fluorescence-activated cell sorting (FACS).

### Luciferase-Based Cytotoxicity Assay

Tumor cells were plated in RPMI 1640-based complete media (see above) at 50,000 cells per well in 96-well flat bottom white plates (Greiner bio one). Synthetic D-luciferin (Sigma Aldrich) was added at 4 µg/ml. Effector cells and/or patient sera were plated at an effector to target ratio (E:T) of 5:1. The total volume per well was 200 µl. GD2-mAb ch14.18/CHO was used at 1 µg/ml unless indicated otherwise. Plates were incubated in a HERAcell incubator (Heraeus) at 37°C, 95% humidity and 5% CO_2_. Plates were measured using the Wallac Victor 1420 Multilabel Counter (Perkin Elmer) at 37°C after 24 h. Lysis was determined by the relative luminescence of the testing condition to a dilution series of target cells (100, 75, 50, 25, 10, and 0%) according to standard controls.

### Sample Collection for FcGR Polymorphisms and KIR Genotyping

EDTA-anticoagulated whole blood samples (5–10 ml) were collected from stem cell donors before haploidentical SCT at the University of Tuebingen, Germany. DNA was extracted using standard methodologies based on spin column technologies (Qiagen) and frozen at −20°C until further analysis.

### Analysis of FcGR Polymorphisms

The analysis of FcGR3A 158-F/V (rs396991) was carried out as proposed by Dall’Ozzo and colleagues (22). Briefly, 5 µl of Sybr Mix was added to 2 µl of PCR-grade water (Peqlab), 1 µl of genomic DNA [final concentration: 10 ng], and 1 µl of FcGR specific primers [each 5 pmol], respectively. After an initial denaturation step for 1 min at 95°C, 35 PCR cycles of 3 s at 95°C and 20 s at 59°C were run on the CFX96 Real-Time PCR Detection System (Biorad). By post-amplification melting curve analysis, the V allele (melting point 83°C) and F allele (melting point 88°C) were distinguished. Restriction fragment length polymorphism (RFLP) assays were applied to analyze FcGR2A 131-H/R (rs1801274) polymorphism (Jiang et al., 1996). A 366 bp region was amplified by GoTaq DNA Polymerase (Promega), digested with BstUI (H/H 343 bp and R/R 322 bp), and separated by agarose gel electrophoresis.

## Statistics

For statistical analysis GraphPad Prism 8.4.3 (GraphPad Software Inc., La Jolla/CA, USA) was used. For comparing two groups, the t-test was used. For comparing three or more groups, the one-way-ANOVA test and *post-hoc* Tukey were used. P-values below 0.05 were defined significant.

## Results

In this study, we examined patients with histologically confirmed Stage IV neuroblastoma at relapse post standard therapies, who were treated between 2010 and 2017 in a prospective multicenter Phase I/II trial (NCT02258815) with a combination of haploidentical HSCT and consecutive GD2 dinutuximab beta (ch14.18) mAb therapy administered with IL-2. Conditioning regimen included fludarabine (40 mg/m²), thiotepa (10 mg/kg), melphalan (70 mg/m²) as well as anti-thymocyte globulin (ATG, Fresenius) 30 mg/kg on days −12 to −9. Grafts were T- and B-cell depleted by CD3 and CD19 *via* magnetic-activated cell sorting from G-CSF-mobilized apheresis from haploidentical donors, as previously described (14, 23). Mycophenolate mofetil (1,200 mg/m²/day) was applied as posttransplant GVHD-prophylaxis until day +30 if residual T cells in the graft exceeded 2.5 × 10^4^/kg BW. GD2 mAb therapy was initiated between day +60 and day +180 posttransplant if patients showed no signs of GvHD and required no immunosuppressive medications. The protocol consisted of six consecutive 4-week cycles at 20 mg/m^2^ dinutuximab beta (ch14.18/CHO), which was administered as a continuous intravenous infusion over a period of 8 h per day on the first 5 days of each cycle. IL2 (Aldesleukin) was administered during the cycles 4 to 6 on the days +6, +8 and +10 of the corresponding cycle at 1 × 10^6^ IU/m²/d subcutaneously (s.c.), only in patients with no signs of severe acute GvHD (Grades 3–4) or extensive chronic GvHD. Clinical details will be described in a separate publication.

### Immune Reconstitution Post Haploidentical HSCT, Dinutuximab Beta Serum Levels, and the Development of Neutralizing Human Anti-Chimeric Antibodies

**Figure 1:**
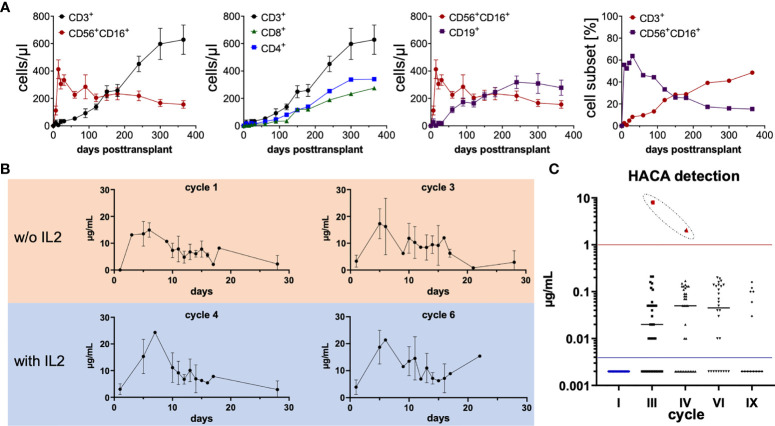
Lymphoid immune reconstitution posttransplant, ch14.18/CHO patient serum levels as well as serum levels of human anti-chimeric antibodies (HACA). **(A)** Posttransplant lymphoid immune reconstitution of CD3^+^ T cells (CD4^+^ and CD8^+^), CD19^+^ B cells, and CD56^+^CD16^+^ NK cells was assessed by flow cytometry at indicated time points. A total of n = 36 eligible patients were included in the analysis. Absolute cell counts per microliter blood (mean ± SEM) were calculated from flow cytometric frequencies (%) and total lymphoid cells derived from the whole blood counts of patients. In the right panel the mean frequencies [%] of CD3^+^ and CD56^+^CD16^+^ are shown. **(B)** ch14.18/CHO patient serum levels at indicated time points during cycles 1, 3, 4, and 6 were measured by triple-ELISA as previously described (15). A minimum of one and a maximum of n = 15 in cycle 1, n = 14 in cycle 3, n = 12 in cycle 4, and n = 13 in cycle 6 serum of patients were measured. Basically in all ch14.18/CHO treatment cycles, a continuous relevant serum concentration above 1 µg/ml was sustained, facilitating a strong immunologic anti-tumor effect and consolidation by complement-dependent and cellular-dependent cytotoxicity throughout the course of antibody therapy (>6 months). **(C)** Neutralizing HACA antibodies were measured by ELISA as previously described (19). Only in one patient (red dots), HACAs were detected at relevant levels, indicated by the red horizontal line, in cycle 3 and cycle 4. No further data for this patient is available since no additional testing was performed in this patient afterwards. In cycle 1 n = 32, cycle 3 n = 37, cycle 4 n = 36, cycle 6 n = 28, cycle 9 n = 14 patients were evaluated. In order to display the 0 values in the logarithmic scale, 0 was substituted by 0.002 below the detection threshold (indicated by the blue horizontal line).

To assess the requirements for cooperative antitumoral immune activation, as envisioned in the study design, immune reconstitution as well as pharmacokinetics of dinutuximab beta was monitored. A total of n = 36 eligible patients were included in the analysis. Absolute cell counts per microliter blood (mean ± SEM) were calculated from flow cytometric frequencies (%) and total lymphoid cells derived from the patients’ whole blood counts. Haploidentical HSCT was followed by rapid NK-cell reconstitution. The “NK cell wave” peaked at day +14 posttransplant with a median cell count of 413 (108 to 1,424) CD56^+^CD16^+^ cells/µl in the peripheral blood. T-, B- and NK-cell reconstitution was within the expected ranges for CD3/CD19 depleted grafts, with a median of 256 (34 to 923) CD3^+^, 120 (13 to 396) CD4^+^ and 140 (6 to 555) CD8^+^ as well as 246 (61 to 771) CD19^+^ cells/µl, and 423 (32 to 1,278) CD56^+^ cells/µl at 6 months posttransplant and full recovery at the first year after haploidentical HSCT in most patients. The time point of T cells representing the main lymphoid population was reached at approximately day +150, as demonstrated in **Figure 1A** by absolute numbers (cells/µl) and % cell subsets for T cells and NK cells (**Figure 1A**).

Administration of dinutuximab beta resulted in sufficient serum levels, peaking at approximately day 5 of each cycle, after a total infusion of 100 mg/m^2^ was completed. Serum levels were measured by triple-ELISAs as previously described (15). A minimum of 1 and a maximum of n_cycle 1_ = 15, n_cycle 3_ = 14, n_cycle 4_ = 12 and n_cycle 6_ = 13 patient sera were measured. The serum levels of ch14.18/CHO remained above the *ex vivo* evaluated effective concentration >1 µg/ml over the course of the protocol until all cycles were completed for cycles 1–6 (**Figure 1B**). As some patients even received nine cycles of dinutuximab beta, it is most likely that these patients achieved a clinically relevant serum concentration of ch14.18/CHO for a total of 9 months as an immunologic antitumor consolidation treatment posttransplant, which could strongly mediate both antibody-dependent cellular cytotoxicity (ADCC) as well as complement-dependent cytotoxicity (CDC).

Since dinutuximab beta is a chimeric mAb, patients were monitored for human anti-chimeric antibody (HACA), capable of neutralizing therapeutic activity of mAbs. Neutralizing HACA antibodies were measured by ELISAs as previously described (19). There were no detectable HACA prior to initiation of ch14.18/CHO treatment in all evaluated patients. Solely in one patient, indicated by red dots, HACAs were detected at relevant levels in cycle 3 at 8 µg/ml and cycle 4 at 2 µg/ml. No further data on the course of HACA is available for this patient. The following number of patients was tested during the indicated cycles— n_prior to cycle 1_ = 32, n_cycle 3_ = 37, n_cycle 4_ = 36, n_cycle 6_ = 28, n_cycle 9_ = 14 (**Figure 1C**).

### Elevation of Proinflammatory Cytokines and NK Cell Activation as a Consequence Of Dinutuximab Beta Immunotherapy

**Figure 2:**
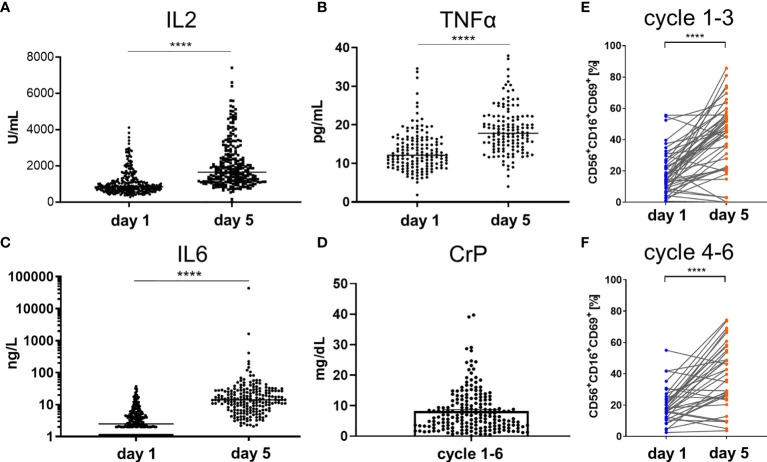
Elevation of proinflammatory cytokines and activation of NK cells by ch14.18/CHO antibody infusion therapy. The infusion of ch14.18/CHO induced robust immune response measured by means of secretion of proinflammatory cytokines **(A)** IL2, **(B)** TNFα, **(C)** IL6, and **(D)** CrP in serum of patients or lithium heparin plasma as well as by the percentage increase in number of activated NK cells **(E, F)**. Cytokines and NK cells were measured on day 1 prior to the start of antibody infusion and on day 5 of cycles 1 to 9. The maximum level of CrP per cycle is shown for the cycles 1–6 in **(D)**. Every single dot represents an independent single value per cycle and patient used from cycles 1–6 **(A–D)** but as indicated in **(E)** cycles 1–3 and **(F)** cycles 4–6. For the comparison of **(A)** IL2 n = 232, **(B)** TNFα n = 137, **(C)** IL6 n = 207 was available in pair values and for **(D)** CrP n = 198 single values were used. NK cell immunophenotype was assessed by flow cytometry and was defined as the CD56^+^CD16^+^ and CD3^−^ subset of lymphoid cells. The early activation marker CD69 was used to distinguish resting (CD69^−^) from activated (CD69^+^) NK cells n_cylces 1–3_ = 47, n_cylces 4–6_ = 36. Statistical analysis was done by two-tailed paired t-test. P-values below 0.05 were defined significant. **** = <0.0001.

To demonstrate the functional activity of the newly established haploidentical immune system in combination with GD2 mAb therapy, biomarkers for inflammation and NK cell activation were monitored. Administration of dinutuximab beta resulted in a highly significant and temporally cohesive induction of the proinflammatory cytokines measured on day 1 prior to start of ch14.18/CHO infusion and on day 5 of any treatment cycle (**Figure 2**). In general, patient values for the assessed inflammatory markers were above normal values on day 5. The number of value pairs corresponded to the treatment cycles ranging from one up to a maximum of nine cycles per patient. The means of soluble IL2 receptors on day 1 (IL2^day 1^) and on day 5 (IL2^day 5^) were respectively 1,062 IU/ml (range 284–4,109) and 2,046 (range 11–7,406) [standard value 300–900 IU/mL] n_IL2_ = 232 (p < 0.0001) (**Figure 2A**). The mean of TNFα^day 1^ was 13.3 pg/ml (range 1.8–34.6 pg/ml), *versus* TNFα^day 5^ at 18.5 (range 4–37.90 pg/ml) [standard value 0–25 IU/ml] n_TNFα_ = 127 (p < 0.0001) (**Figure 2B**). The mean of IL6^day 1^ was 4.7 pg/ml (range 1.1–37.1 pg/ml) *versus* IL6^day 5^ at 241 pg/ml (range 2.1–43,469 pg/ml) [standard value < 5 pg/ml] n_IL6_ = 207 (p < 0.0001) (**Figure 2C**). The mean maximum CrP value per cycle was 8.2 mg/dl (range 0.1–39.71 mg/dl) [standard value < 0.5 mg/dl] n_CrP_ = 198 (p < 0.0001) (**Figure 2D**). Analysis of NK cells freshly isolated from patient peripheral blood demonstrated a significant increase in the expression of the activation marker CD69 on NK cells during the course of treatment from day 1 to day 5 of dinutuximab beta (n_cylces 1–3_ = 47, p < 0.0001; n_cylces 4–6_ = 36, p < 0.0001). This increase was especially prominent in CD16^+^ NK cells, indicative of stimulation *via* Fc-binding as a surrogate for active ADCC (**Figures 2E, F**). Statistical analyses were performed by a two-tailed paired t-test on the paired values of one patient.

### Comparison of Early (Cycles 1–3) *Versus* Later (Cycles 4–6) Dinutuximab Beta Treatment Cycles and the Impact Of Subcutaneous IL2 Application on Proinflammatory Cytokines and NK Cell Activation State

**Figure 3:**
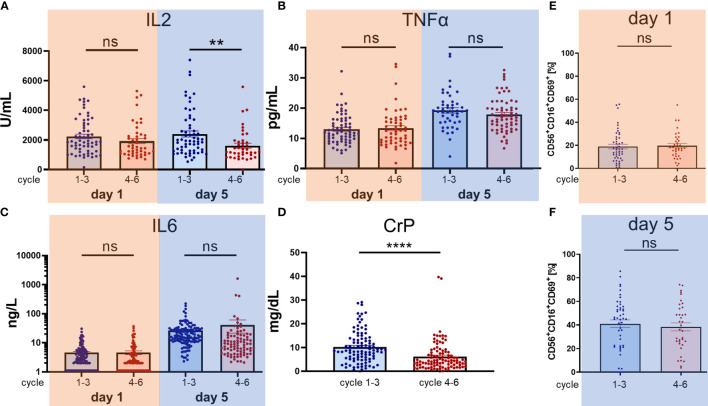
Comparison of cytokine secretion and activation of NK cells during cycles 1–3 without IL2 administration and cycles 4–6 with low dose subcutaneous IL2 administration. In contrast to cycles 1–3 starting as early as 60 days post haploidentical HSCT, in cycles 4–6 at days 6, 8, and 10 subcutaneous IL2 is administered at 1E06 IU/m^2^/day. Consequently, we performed a systematic analysis and comparison of cytokine secretion and the activation of NK cells between the cycles 1–3 *versus* the cycles 4–6. We compared the levels of the proinflammatory cytokines **(A)** IL2, **(B)** TNFα, **(C)** IL6 and **(D)** CrP in serum of patients or lithium heparin plasma and the percentage increase in number of activated NK cells **(E, F)**. Every single dot represents an independent single value per cycle and patient used from cycles 1–6 **(A–D)** but as indicated in **(E)** cycles 1–3 and **(F)** cycles 4–6. For the comparison of **(A)** IL2 day 1 n_cycles 1–3_ = 62/n_cycles 4–6_ = 46, day 5 n_cycles 1–3_ = 62/n_cycles 4–6_ = 41, **(B)** TNFα day 1 n_cycles 1–3_ = 67/n_cycles 4–6_ = 55, day 5 n_cycles 1–3_ = 63/n _cycles 4–6_ = 47, **(C)** IL6 day 1 n_cycles 1–3_ = 129/n_cycles 4–6_ = 97, day 5 n_cycles 1–3_ = 125/n_cycles 4–6_ = 86 was available in pair values and for **(D)** CrP n = 99 single values were used. NK cell immunophenotype was assessed by flow cytometry and was defined as the CD56^+^CD16^+^ and CD3^−^ subset of lymphoid cells. The early activation marker CD69 was used to distinguish resting (CD69^−^) from activated (CD69^+^) NK cells, day 1 and 5 n_cylces 1–3_ = 47, n_cylces 4–6_ = 36, day 1 p = 0.78, day 5 p = 0.58. Statistical analysis was done by two-tailed unpaired t-test. P-values below 0.05 were defined significant. ** = <0.01, **** = <0.0001. ns, not significant.

Notably, neither an increase in inflammatory cytokines including IL2, TNFα, IL6, CrP, nor an increase of NK-cell activation CD69^+^ positivity was observed when comparing cycles 1 to 3 (without s.c. IL2 application) to cycles 4 to 6 (with additional s.c. IL2 application) as illustrated in **Figure 3**. For the comparison between the treating conditions without IL2 (cycles 1–3) and with IL2 s.c. application (cycles 4–6), the data from cycles 7–9 were excluded. The difference for IL2 (**Figure 3A**) on day 1 was not significant (n_cycles 1–3_ = 62 *vs.* n_cycles 4–6_ = 46, p = 0.17), but on day 5 the sILR level was significantly higher during cycles 1–3 without s.c. IL2 application (n_cycles 1–3 =_ 62/n_cycles 4–6_ = 41, p = 0.0057). There was no difference in TNFα levels on day 1 (n_cycles 1–3_ = 67/n_cycles 4–6_ = 55, p = 0.71) or day 5 (n_cycles 1–3_ = 63/n_cycles 4–6_ = 47, p = 0.24) (**Figure 3B**). Further, no difference was found in IL6 levels (**Figure 3C**) on day 1 (n_cycles 1–3_ = 129/n_cycles 4–6_ = 97, p = 0.99) or on day 5 (n_cycles 1–3_ = 125/n_cycles 4–6_ = 86, p = 0.39). In line with higher sILR levels in cycles 1–3 compared to cycles 4–6, there was a significant increase in CrP (n_cycles 1–3_ = 99, n_cycles 4–6_ = 99, p < 0.0001) (**Figure 3D**). NK cell immunophenotype was assessed by flow cytometry and was defined as the CD56^+^CD16^+^ and CD3^−^ subset of lymphoid cells (**Figures 3E, F**). The early activation marker CD69 was used to distinguish resting (CD69^−^) from activated (CD69^+^) NK cells, with days 1 and 5 n_cylce 1–3_ = 47, n_cylces 4–6_ = 36, day 1 p = 0.78, day 5 p = 0.58. Statistical analysis was done by two-tailed unpaired t-tests for IL2, TNFα, IL6, and NK activation marker CD69^+^. Only the daily evaluated laboratory marker CrP was available in a complete data set to perform a paired analysis using the paired t-tests. P-values below 0.05 were defined as significant.

### Evaluation of NK Cell Mediated ADCC and CDC Utilizing Dinutuximab Beta

#### Degranulation of NK Cells (CD107a Assay)

**Figure 4:**
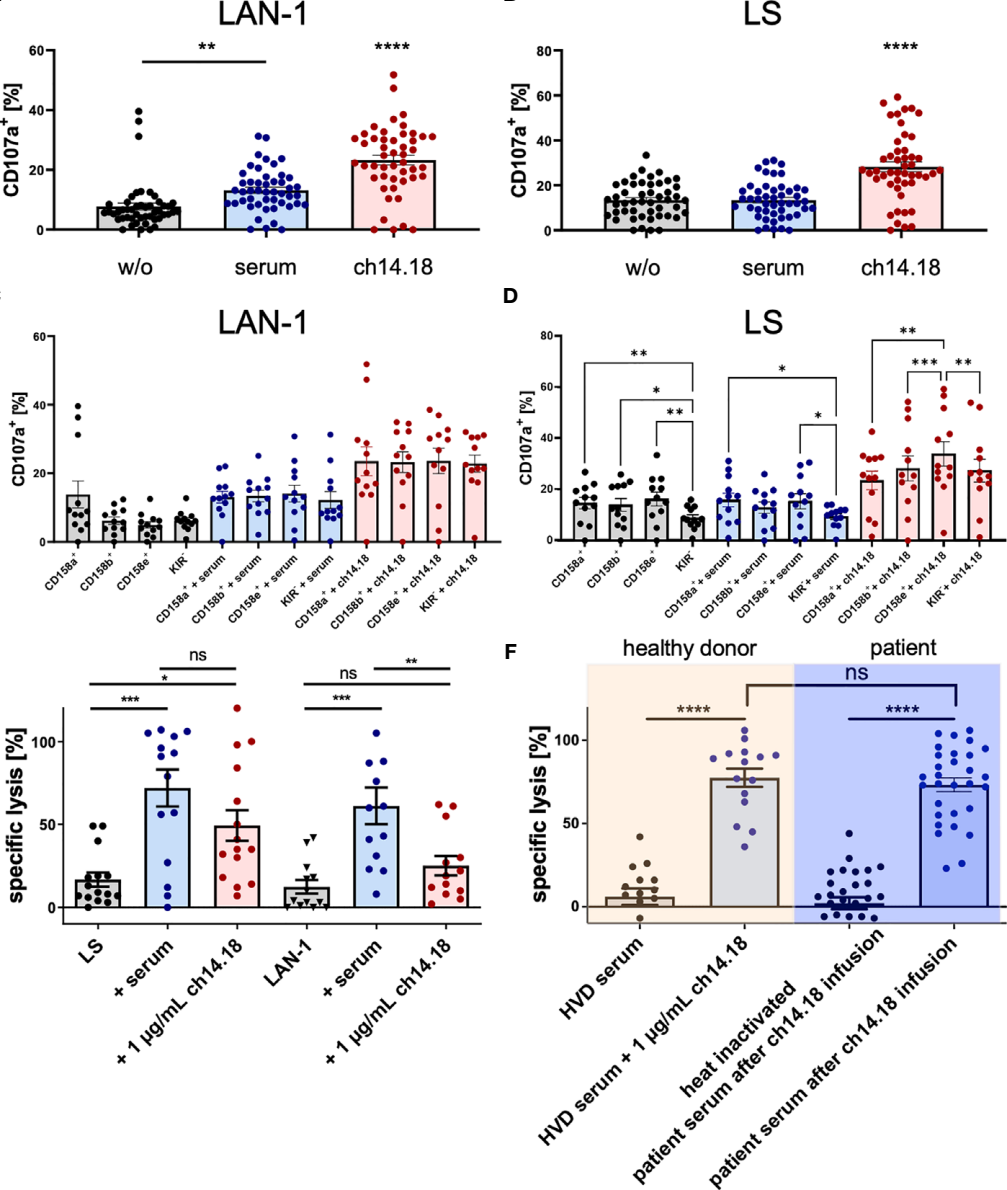
Degranulation of patient NK cells and cytolysis mediated by ch14.18/CHO. Patient PBMCs acquired post haploidentical HSCT were used to test the capacity of degranulation, measuring CD107a (LAMP-1) expression as a strong indicator of NK cell activation and cytolysis targeting the neuroblastoma cell lines LAN-1 and LS in a 6 h flow cytometry based kill assay **(A–D)**. Besides testing the overall degranulation capacity, NK cell subsets distinguished by the major inhibitory killer cell immunoglobulin-like receptors (KIR) CD158a/KIR2DL1, CD159b/KIR2DL2 and CD158e/KIR3DL1 were analyzed for interpopulation differences in CD107a positivity and were evaluated in the context of the KIR receptor ligand model. Additionally, a 2 h-DELFIA-EuTDA release cytotoxicity assay was used to evaluate the cytolysis of tumor cells by patient PBMCs *via* ADCC and CDC **(E, F)**. The testing conditions **(E)** comprised I) PBMCs *versus* tumor, II) PBMCs *versus* tumor and patient serum (after infusion of ch14.18/CHO), and III) PBMCs *versus* tumor and 1 µg/ml ch14.18/CHO. Moreover, cytolysis of the neuroblastoma cell line LAN-1 by PBMCs from healthy volunteer donors (HVDs) compared to patient PBMCs was assessed. **(F)** The comparison included the conditions I) [HVD PBMCs plus HVD serum] *versus* tumor, II) [HVD PBMCs plus HVD serum plus 1 µg/ml ch14.18/CHO] *versus* tumor, III) [patient PBMCs plus heat inactivated patient serum] *versus* tumor, and IV) [patient PBMCs plus patient serum] *versus* tumor. Data shown in **(A–D)** represent mean of (n = 12) independent experiments and different donors in triplicates, respectively. Data shown in **(E)** represent mean of (n_LAN-1_ = 15; n_LS_ = 13) and **(F)** represent single values of (n_HVD_ = 5; n_patients_ = 10) independent experiments and different donors in triplicates, respectively. Statistical significance was determined by one-way ANOVA and Tukey *post-hoc* test. P-values below 0.05 were defined significant. * = <0.05, ** = <0.01, *** = <0.0001, **** = <0.0001. ns, not significant.

NK cell degranulation and cytolytic activity are shown in **Figure 4**. To prove the specific cytolytic activity of NK cells recruited to tumor cells by dinutuximab beta as a result of ADCC and CDC, patient derived-PBMCs acquired post haploidentical HSCT and patient sera post dinutuximab beta infusion and the condition 1 µg/ml ch14.18/CHO were analyzed *ex vivo*. Specific activities against two established neuroblastoma cell lines LS and LAN-1 were assessed. Patient-derived PBMCs were co-cultured with target cells in the presence of patient serum or dinutuximab beta at 1 µg/ml. First, degranulation, measured by CD107a (LAMP-1) positivity as a strong indicator of NK cell activation and cytolysis, by patient-derived PBMCs in the presence of patient serum or dinutuximab beta was analyzed in a 6 h flow cytometric based kill assay. The results demonstrated significant increases in cytolysis in the conditions with GD2 antibody in the presence of patient serum and 1 µg/ml ch14.18/CHO for the cell line LAN-1, but only in the condition with 1 µg/ml ch14.18/CHO for the cell line LS [**Figures 4A–D** n = 12 independent experiments and donors; p_LAN-1_ = 0.0112 (without mAb *versus* serum), p_LAN-1_ < 0.0001 (1 µg/ml ch14.18/CHO *versus* serum *versus* without mAb). p_LS_ > 0.99 (without mAb *versus* serum), p_LS_ < 0.0001 (1 µg/ml ch14.18/CHO *versus* without mAb and *versus* serum)] (**Figures 4A, B**).

Moreover, the single inhibitory killer cell immunoglobulin-like receptor (KIR) positive NK cell subsets CD158a/KIR2DL1, CD159b/KIR2DL2, and CD158e/KIR3DL1 were compared with regard to the overall degranulation capacity to address the impact of the KIR receptor ligand model (KIR R/L) in neuroblastoma. The comparison of NK subsets *versus* LAN-1 revealed no significant differences within the groups (p_w/o mAb_ = 0.60, p_serum_ = 0.93, p_ch14.18_ = 0.08) (**Figure 4C**). In **Figure 4D**, the comparison of NK subsets *versus* LS was illustrated with a significant difference found—p_w/o mAb_ = 0.0006 (in the *post-hoc* test p_CD158a+_ *_vs_*_. KIR−_ = 0.0092, p_CD158b+_ *_vs._*_KIR−_ = 0.39, p_CD158e+_ *_vs._*_KIR−_ = 0.0068), p_serum_ = 0.0028 (in the *post-hoc* test p_CD158a+_ *_vs_*_. KIR−_ = 0.0131, p_CD158e+_ *_vs_*_. KIR−_ = 0.0489), p_ch14.18_ = 0.0004 (in the *post-hoc* test p_CD158a+_ *_vs_*_. CD158e+_ = 0.003, p_CD158b+_ *_vs_*_. CD158e+_ = 0.0004, p_CD158e+_ *_vs._*_KIR−_ = 0.0082).

#### EuTDA Release Cytotoxicity Assay

Additionally, a 2 h-DELFIA-EuTDA release cytotoxicity assay was used to evaluate the cytolysis of patient PBMCs (**Figures 4E, F**). The testing conditions comprised: I) PBMCs *versus* tumor, II) PBMCs *versus* tumor and patient serum (after infusion of ch14.18/CHO), and III) PBMCs *versus* tumor and 1 µg/ml ch14.18/CHO. Moreover, cytolysis of the neuroblastoma cell line LAN-1 by PBMCs from healthy volunteer donors (HVDs) compared to PBMCs of patients was assessed. In contrast to degranulation, as a marker for NK-cell activation, patient serum and the condition with 1 µg/ml ch14.18/CHO mediated significantly higher lysis with patient-derived PBMCs compared to the condition without mAb (p = 0.0002). The condition patient serum *versus* 1 µg/ml ch14.18/CHO indicated a trend (ns) that complement-dependent cytotoxicity (CDC) contributes to total cell lysis (**Figure 4E**). To further substantiate this observation, specific lysis mediated by patient sera, acquired post dinutuximab beta infusion, was compared to serum of untreated healthy volunteer donors, untouched or substituted with dinutuximab beta, and heat inactivated patient serum. Clearly, specific lyses in the absence of effector cells requires both dinutuximab beta and a functional complement system (p = 0.0001), demonstrating CDC as a substantial mechanism of dinutuximab beta-mediated activity (**Figure 4F**). Data shown in **Figures 4A–D** represent mean of (n = 12) independent experiments and different donors in triplicates, respectively. Data shown in **Figure 4E** represent mean of (n_LAN-1_ = 15; n_LS_ = 13) and **Figure 4F** represent single values of (n_HVD_ = 5; n_patients_ = 10) independent experiments and different donors in triplicates, respectively. Statistical significance was determined by one-way ANOVA and Tukey *post-hoc* test.

### Impact of GD2 Expression on Dinutuximab Beta-Mediated ADCC and CDC

**Figure 5:**
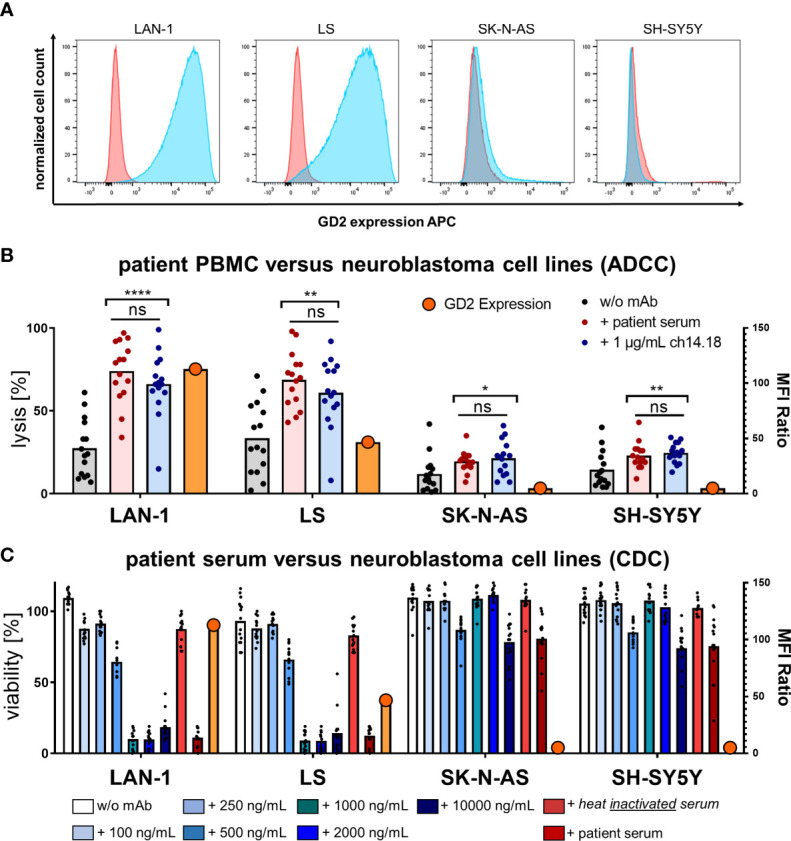
GD2 expression and cytolysis of neuroblastoma cell lines *via* ADCC and CDC by PBMCs and serum of patients. **(A)** GD2 expression of the neuroblastoma cell lines LAN-1, LS, SK-N-AS, and SH-SY5Y was measured by flow cytometry using primary labeled GD2 PE (clone 14G2a) mAb and a mouse IgG2a, k PE mAb as isotype control. The median fluorescence intensity (MFI) was used to calculate the MFI ratio (MFIR) by the MFI GD2 PE mAb divided by MFI isotype control IgG2a, k PE mAb. Representative univariate histograms are shown. From left to right panels the relative GD2 expression declines MFIR LAN-1 = 112.9 > MFIR LS = 46.8 > MFIR SK-N-AS = 1.3 > MFIR SH-SY5Y = 1. **(B)** Patient PBMCs acquired post haploidentical HSCT were used to test the direct cellular cytotoxicity and ADCC at an effector to target ratio E:T 5:1 of PBMCs *versus* the indicated neuroblastoma cell line in a 24 h luciferase-based kill assay. Specific lysis was assessed in the conditions I) PBMCs without mAb *versus* tumor, II) PBMCs plus patient serum *versus* tumor, III) PBMCs plus 1 µg/ml ch14.18/CHO mAb *versus* tumor. **(C)** CDC was tested in a coincubation of human serum from healthy volunteer donors (HVDs) at different levels of concentrations of added ch14.18/CHO mAb or heat-inactivated serum of patients or serum of patients *versus* the indicated neuroblastoma cell lines in a 24 h luciferase-based kill assay without adding any effector cells. Data shown in **(B)** represent mean of triplicates of (n = 15) independent experiments; data shown in **(C)** represent (n = 3) independent experiments of (n = 15) different donors in **(B, C)**, respectively. Statistical significance was determined by one-way ANOVA and Tukey *post-hoc* test. P-values below 0.05 were defined significant. ADCC, antibody-dependent cellular cytotoxicity; CDC, complement-dependent cytotoxicity; HVD, healthy volunteer donor. * = <0.05, ** = <0.01, **** = <0.0001. ns, not significant.

GD2 expression on neuroblastoma tumors may vary individually from patient to patient. To further decipher the contribution of ADCC and CDC to target-antigen specific antitumoral activity, patient-derived PBMCs and sera were studied in 24 h luciferase-based cytotoxicity assays (LCAs) using neuroblastoma cell lines with high (LAN-1 and LS) and low or absent (SK-N-AS and SH-SY5Y) GD2 expression (**Figure 5A**). GD2 expression was measured by flow cytometry using primary labeled GD2 PE (clone 14G2a) mAb and a mouse IgG2a, k PE mAb as isotype control. The median fluorescence intensity (MFI) was used to calculate the MFI ratio (MFIR) by the MFI GD2 PE mAb divided by MFI isotype control IgG2a, k PE mAb. The relative GD2 expression was MFIR LAN-1 = 112.9 > MFIR LS = 46.8 > MFIR SK-N-AS = 1.3 > MFIR SH-SY5Y = 1.

Patient-derived PBMCs acquired post haploidentical HSCT were used to test the direct cellular cytotoxicity and ADCC at an effector to target ratio E:T 5:1. Specific lysis was assessed in the conditions I) PBMCs without mAb *versus* tumor, II) PBMCs plus patient serum *versus* tumor, III) PBMCs plus 1 µg/ml ch14.18/CHO mAb *versus* tumor. In **Figure 5B** PBMCs mediated significantly improved target cell lysis in the presence of dinutuximab beta and patient sera; post dinutuximab beta infusion strictly depended on target-antigen expression (n = 15, p_LAN-1_ < 0.0001, p_LS_ < 0.01, p_SK-N-AS_ < 0.05, p_SH-SY5Y_ < 0.005). The same antigen specificity was found for CDC (**Figure 5C**). CDC was tested in a coincubation experiment of human serum from healthy volunteer donors (HVDs) at different concentration levels of added ch14.18/CHO mAb or heat-inactivated serum of patients or serum of patients *versus* the indicated neuroblastoma cell lines *without* adding any effector cells. ch14.18/CHO antibody titration experiments demonstrated a threshold for CDC induction *ex vivo* at levels of dinutuximab beta approximately 500 ng/ml in the presence of human serum. Importantly, serum levels of dinutuximab beta in patients were more than two log-fold higher during treatment cycles, indicating highly sufficient conditions for CDC in patients, functionally confirmed by patient sera-mediated lysis. Again, heat inactivation prevented effector cell lysis by patient sera, confirming CDC activity. Data shown in (**Figure 5C**) represent (n = 3) independent experiments of (n = 15) different donors. Statistical significance was determined by one-way ANOVA and Tukey *post-hoc* test. ADCC is antibody-dependent cellular cytotoxicity, and CDC is complement-dependent cytotoxicity.

### Impact of Fc*γ* Receptor Polymorphisms on Patient Outcome

In addition to functional patient monitoring, the relevance of genetic preconditions with regard to polymorphisms in the Fc-gamma-receptor (FCGR2A and FCGR3A) genes were studied in the haploidentical transplant setting. Among the 33 donors analyzed, n = 9 (27%) were VV homozygous, n = 10 (30%) were VF, and n = 14 (42%) were FF homozygous for rs396991 (FcGR3A). Analysis of rs1801274 (FcGR2A) revealed n = 5 patients (15%) homozygous for HH, n = 24 (73%) heterozygous for HR, and n = 4 (12%) homozygous for RR. These data are in line with the expected genotype frequencies for European individuals available at NCBI (HapMap-CEU). Patients homozygous for low-affinity polymorphisms were assigned to the low-affinity cohort; patients homozygous for high-affinity polymorphisms or heterozygous were assigned to the high-affinity cohort as previously described (13). Studying the impact of polymorphisms rs1801274 and rs396991 in *FCGR2A* and -*3A* genes on EFS and OS, no statistical association was found, respectively. No association of Fc-gamma-receptor polymorphism and KIR content on survival was found.

## Discussion

Haploidentical HSCT has evolved from being a well-acknowledged treatment procedure in high-risk leukemias in need of an allogeneic stem cell transplantation (23–25) to being an efficacious treatment for Stage IV relapsed high-risk neuroblastoma patients. Long-term remission can be achieved in a proportion of patients with a tolerable side effect profile (14). In case of *ex vivo* graft manipulation procedures (enrichment of stem cells or T-cell depletion), NK cells have been shown to rapidly reconstitute and contribute to the reduced relapse rates in the early posttransplant period (26–28). Further, the combination of the chimeric antibody ch14.18 dinutuximab with the granulocyte–macrophage colony-stimulating factor (GM-CSF) or interleukin-2 (IL2) has demonstrated significantly improved 2-year event-free survival in high-risk neuroblastoma patients (9). In order to maximize the treatment effects, the combination of haplo HSCT and GD2 antibody therapy in neuroblastoma has been evaluated in the clinical trial (NCT02258815) registered at (clinicaltrials.gov).

Here, we report on the immunomonitoring results of our phase I/II study and evaluate the feasibility of haplo HSCT in combination with anti-GD2 antibody (dinutuximab beta) treatment in children with Stage IV relapsed neuroblastoma. Detailed clinical outcomes, including toxicities and survival, are the subject of a distinct publication and will be reported separately. In line with the scientific rationale for this combinatorial approach, haplo HSCT led to a rapid and robust establishment of a functional cellular immune system in this cohort of heavily pretreated patients. Especially NK-cell recovery, a prerequisite for ADCC, was seen early after haplo HSCT, with a peak at approximately day +14 and a significant increase in cell numbers throughout the six cycles of dinutuximab beta therapy, which is consistent with our previous findings in haplo HSCT (14, 29–31). The long-term consolidation treatment with dinutuximab was dosed in the range of regimens with proven objective response rates in high-risk and refractory neuroblastoma patients (9, 32) who continuously showed relevant dinutuximab serum levels over the course of treatment. Despite the risk for the development of neutralizing antibodies under dinutuximab treatment during the use of chimeric antibodies (18), there was only one patient who was tested positive for HACAs in the presented cohort. Besides the quantitative assessment of serum mAb levels, objective functional activity of dinutuximab was measured in patients. Administration of dinutuximab beta led to a highly significant increase in activated NK cells and elevated serum marker of inflammation (IL2, TNFα, IL6, and CrP) which is in line with previous findings in the autologous setting (10). Additional administration of IL-2 neither boosted NK-cell activity nor increased the inflammatory response which may result from the massive cytokine secretion induced by the antibody infusion itself, thus giving no rationale for IL-2 application in subsequent studies, which have been proposed in a trial with continuous long-term infusion of dinutuximab (33).

To complement in-patient monitoring, NK-cell activity was analyzed *ex vivo*. As shown before, dinutuximab enhances NK-cell function (34). This was shown with patient-derived NK cells demonstrating potent cytokine secretion, degranulation, and cytotoxicity in combination with dinutuximab beta or patient serum against GD2+ target cells *ex vivo*, underscoring the combinatorial functionality. No significant correlation between cytotoxic activity and KIR receptor–ligand mismatch was found yet on a genetic level. Erbe et al. have shown contradictory data that KIR receptor and ligand interaction on a genetic level can negatively impact on the clinical outcome in the treatment with dinutuximab (35). In addition to ADCC, CDC was identified as a relevant mechanism of dinutuximab beta-mediated cytotoxicity with a clear correlation to antigen expression (GD2). In the autologous setting, we have previously demonstrated that neuroblastoma patients with high-affinity FCGR2A, -3A and stimulatory KIR2DS2 show higher levels of ADCC and improved event-free survival (13). Since haplo HSCT allows donor selection based on possibly beneficial donor characteristics, survival data were correlated to polymorphisms in *FCGR2A* and -*3A* genes as well as KIR-gene content score (36). In contrast to the LTI study (13), in our cohort, haplo HSCT using donors with high affinity FcGR3A or FcGR2A did not have any impact on event-free survival and overall survival in neuroblastoma Stage IV relapsed patients with subsequent GD2 targeted ch14.18/CHO antibody therapy. Further, KIR content did not have an impact on patient outcome. Since no correlation was found, no recommendations for donor selection based on the evaluated variables are possible at this stage. As mentioned above, the impact of the KIR receptors and ligand interaction appears to be unsolved (35).

In summary, we can state that haplo HSCT in combination with targeted immunotherapy utilizing dinutuximab is feasible. Haplo HSCT can serve as a safe and reliable tool to strengthen the cellular immune system in heavily pretreated patients. Moreover, haplo HSCT can provide a platform for cellular immunotherapy, generating highly functional effector cells for NK- or T-cell based therapies. This study has important implications for the future therapy of neuroblastoma patients.

## Data Availability Statement

The original contributions presented in the study are included in the article/supplementary material. Further inquiries can be directed to the corresponding author.

## Ethics Statement

The studies involving human participants were reviewed and approved by University of Tuebingen. Written informed consent to participate in this study was provided by the participants’ legal guardian/next of kin.

## Author Contributions

CM: interpretation of the data, drafted the manuscript, and approved the final revision. TF, A-ML, MM, SM, MK, DS, AJ, AR, NS, ST-M, MZ, HL, DA, A-SM, SS, and FH: acquisition, analyzed and interpreted the data, and approved the final revision. SY and RH analyzed and interpreted the data, and approved the final revision. PL: designed study, analyzed and interpreted the data, drafted the manuscript, and approved the final revision. PS: designed study, acquisition, analyzed and interpreted the data, drafted the manuscript, and approved the final revision. All authors contributed to the article and approved the submitted version.

## Funding

This study was supported by grants from the Deutsche Kinderkrebsstiftung (DKS 2014.05) Bonn, Germany to PL and HL; from the Dieter Schwarz Stiftung Neckarsulm, Germany, from the Reinhold-Beitlich Stiftung Tuebingen, Germany, Gesellschaft für Kinderkrebsforschung, Geltendorf, Germany, and from the Stiftung fuer krebskranke Kinder Tuebingen e.V. Germany,to P.L., and from the excellence cluster iFIT (EXC 2180) [Gefördert durch die Deutsche Forschungsgemeinschaft (DFG) im Rahmen der Exzellenzstrategie des Bundes und der Länder – EXC 2180 – 390900677 to PL and PS. We thank the Foerderverein fuer krebskranke Kinder Tuebingen e.V. Germany for continuous support.

## Conflict of Interest

The authors declare that the research was conducted in the absence of any commercial or financial relationships that could be construed as a potential conflict of interest.
